# Sds22, a PP1 phosphatase regulatory subunit, regulates epithelial cell polarity and shape [Sds22 in epithelial morphology]

**DOI:** 10.1186/1471-213X-9-14

**Published:** 2009-02-19

**Authors:** Felix A Grusche, Cristina Hidalgo, Georgina Fletcher, Hsin-Ho Sung, Erik Sahai, Barry J Thompson

**Affiliations:** 1 European Molecular Biology Laboratory, Meyerhofstrasse 1, Heidelberg, Germany; 2 Cancer Research UK London Research Institute, 44 Lincoln's Inn Fields, London, UK; 3 Peter Macallum Cancer Research Centre, St Andrews Place, East Melbourne, Victoria, Australia

## Abstract

**Background:**

How epithelial cells adopt their particular polarised forms is poorly understood. In a screen for genes regulating epithelial morphology in *Drosophila*, we identified *sds22*, a conserved gene previously characterised in yeast.

**Results:**

In the columnar epithelia of imaginal discs or follicle cells, mutation of *sds22 *causes contraction of cells along their apical-basal axis, resulting in a more cuboidal morphology. In addition, the mutant cells can also display altered cell polarity, forming multiple layers in follicle cells and leaving the epithelium in imaginal discs. In yeast, *sds22 *encodes a PP1 phosphatase regulatory subunit. Consistent with this, we show that *Drosophila *Sds22 binds to all four *Drosophila *PP1s and shares an overlapping phenotype with *PP1beta9c*. We also show that two previously postulated PP1 targets, Spaghetti Squash and Moesin are hyper-phosphorylated in *sds22 *mutants. This function is shared by the human homologue of Sds22, PPP1R7.

**Conclusion:**

Sds22 is a conserved PP1 phosphatase regulatory subunit that controls cell shape and polarity.

## Background

Epithelial tissues are composed of polarised cells connected by adherens junctions to form continuous sheets with apical and basal surfaces. How epithelial cells maintain their polarity, adhesion and shape remains poorly understood. Polarity in epithelia is founded on the segregation of determinants into apical and baso-lateral membrane domains. Adherens junctions are located at the interface of these domains and connect the actin cytoskeleton to neighbouring cells. Actin filaments are visible around the entire plasma membrane, but a particularly prominent belt of actin filaments runs around the apical cortex, overlapping with the ring of adherens junctions. This apical contractile bundle of actin filaments is likely to be a critical element in organising the polarised form of epithelial cells (reviewed in [1]). Molecules regulating the spatial organisation of the actin cytoskeleton and generation of forces upon it are therefore of particular interest.

Erzin-Radaxin-Moesin (ERM) proteins link actin filaments with the plasma membrane and are necessary to organise the cortical actin cytoskeleton (reviewed in [2]). *Drosphila *has a single ERM family member, Moesin, that is essential for maintenance of epithelial cell polarity and shape. Cells lacking Moesin are unable to maintain their polarised form, disassemble adherens junctions and leave the epithelium, ultimately undergoing apoptosis [3].

Myosin II can slide two actin filaments against each other to create tension (reviewed by [4]. This is the basis for muscle contraction in skeletal muscle, but also has important force-generating roles in non-muscle cells. *Drosophila *has a single non-muscle myosin II heavy chain encoded by *zipper (zip) *and a single non-muscle myosin II regulatory light chain (MRLC) encoded by *Spagetti-Squash (Sqh)*. Analysis of mutant alleles of these genes has revealed that non-muscle myosin II is required for maintenance of epithelial cell shape, as well as other processes involving dynamic cell shape changes such as gastrulation movements and cytokinesis [5-9].

Both Moesin and Myosin II are activated by phosphorylation and concentrated at the apical membranes of *Drosophila *epithelial cells. Several kinases that phosphorylate Moesin (Slik kinase; [10]) and Sqh/MRLC (Rho kinase; see for example [9] have been identified). One of the four *Drosophila *PP1 phosphatases, PP1β9c, has been shown to antagonise Sqh/MRLC phosphorylation [11]. Here, we identify Sds22, a PP1 phosphatase regulatory subunit, that binds to all four *Drosophila *PP1 phosphatases and restricts the activity of both Sqh/MRLC and Moesin. We show that loss of Sds22 has a similar but stronger phenotype than loss of PP1β9c, disrupting both epithelial cell shape and polarity.

## Results

### *sds22 *is required for epithelial morphology in imaginal disc epithelia

In a PiggyBac transposon-mutagenesis screen in the *Drosophila *eye imaginal disc, we recovered an insertion immediately upstream of the start codon of *sds22 *(PB1173) which disrupts epithelial morphology, causing lethality (Additional Fig 1). The lethality and mutant phenotypes of this allele were reverted when the PiggyBac transposon was excised. The PiggyBac transposon allows transcription (data not shown) but is expected to prevent translation of the *sds22 *mRNA. The *sds22*^PB1173 ^phenotype was also rescued by expression of a *UAS.sds22-GFP *transgene (Additional Fig 2). Finally, expression of a UAS.sds22-IR transgenic RNAi line (VDRC 11788) produced phenotypes highly similar to that of *sds22*^PB1173 ^(data not shown). Although we have not specifically established that sds22 expression was reduced or absent in the insertion mutant, we presume that this is the case as the mutant is rescued by an *sds22 *transgene and phenocopied by Sds22-RNAi. Together, these results show that the observed phenotypes are caused by disruption of the *sds22 *gene.

**Figure 1:**
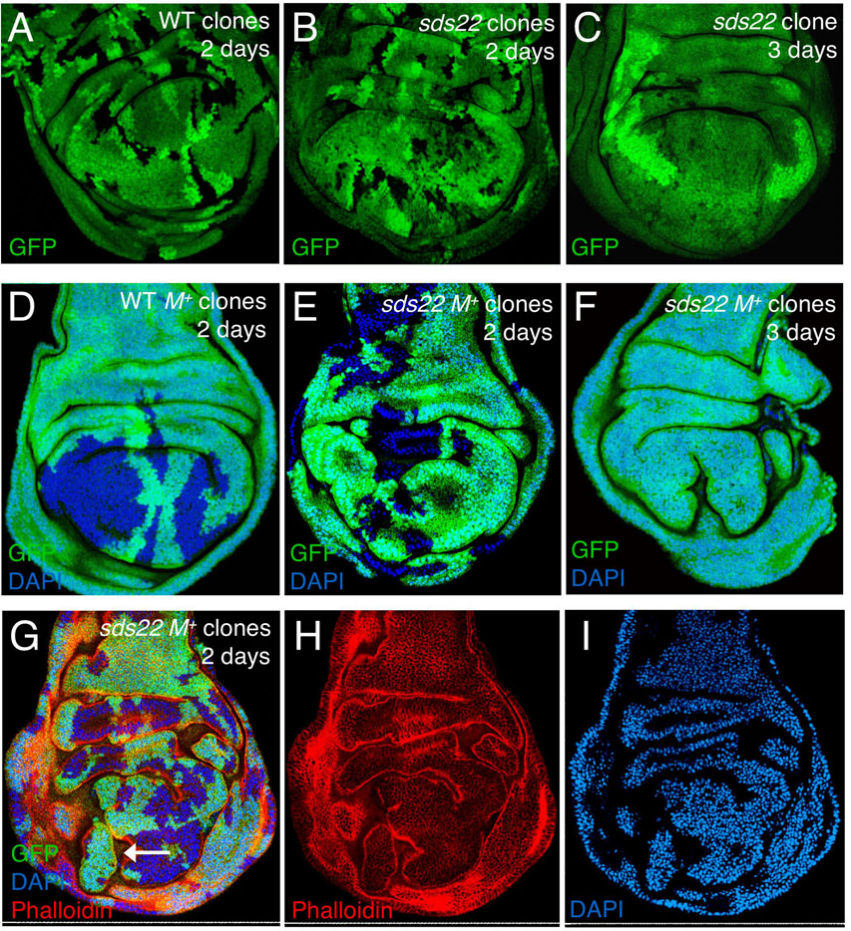
***sds22 *is required to maintain epithelial morphology in imaginal discs**. Mitotic recombination clones were induced in developing wing imaginal discs with the *hs.FLP *system and marked by the absence of GFP. (A) Wildtype clones induced 84 hrs after egg laying (AEL) and allowed to grow for 2 days. (B) *sds22 *mutant clones induced 84 hrs AEL grow to a similar size as wildtype clones after 2 days. (C) *sds22 *mutant clones induced 60 hrs AEL are largely eliminated after 3 days. (D) Wildtype clones induced in a Minute background at 84 hrs AEL grow to large sizes after 2 days. DAPI marks nuclei. (E) *sds22 *mutant clones in a Minute background induced at the same developmental stage (84 hrs AEL) grow to smaller sizes and exhibit morphological defects after 2 days. DAPI marks nuclei. (F) *sds22 *mutant clones induced earlier in development (60 hrs AEL) and allowed to grow for 3 days are eliminated and often leave ectopic folds behind. DAPI marks nuclei. (G) *sds22 *mutant clones induced at 84 hrs AEL and allowed to grow for 2 days. The morphogenesis defect in mutant clones was visualised by staining for phalloidin and DAPI. The arrow points to an island of wild-type tissue surrounded by deeply folded *sds22 *mutant tissue. (H) Phalloidin channel of (G). (I) DAPI channel of (G).

**Figure 2:**
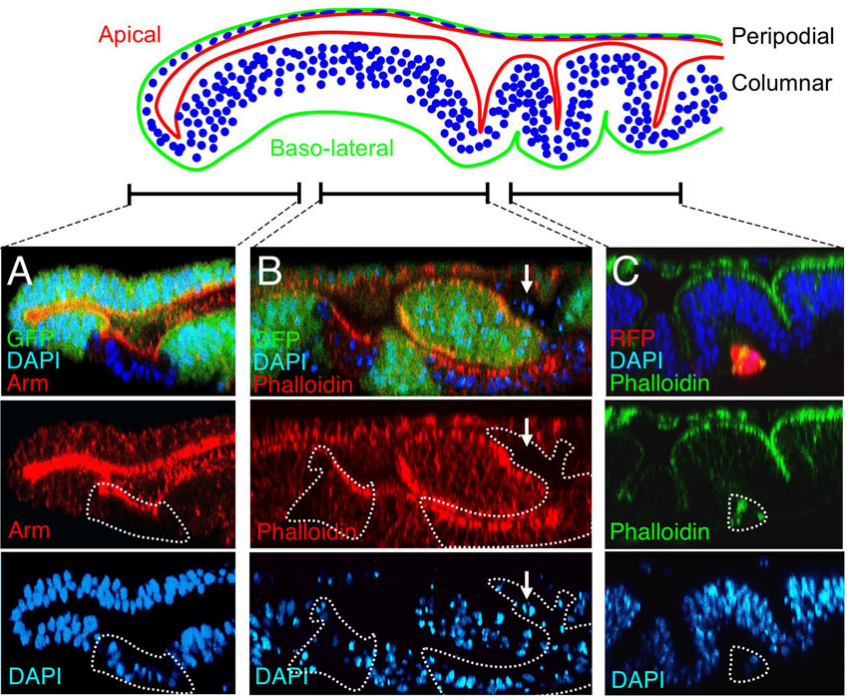
***sds22 *mutant cells change shape and are extruded from imaginal disc epithelia**. Confocal cross-sections through wing discs containing *sds22 *mutant clones marked by the absence of GFP (A and B) or by the presence of RFP (C). DAPI (blue) marks nuclei. Phalloidin or Armadillo strongly labels apical cell junctions and more weakly labels cell outlines. TOP: A diagram of a wing imaginal disc in cross section. (A) A disc containing a small *Minute*+ clone of *sds22 *mutant cells. Mutant cells change shape, shortening along their apical-basal axis and assuming a more cuboidal morphology. In wild-type tissue, nuclei are positioned in a pseudostratified manner, while in mutant tissue nuclei are aligned in a more linear fashion. Note that mutant cells retain correct localisation of the apical adherens junction marker Armadillo. (B) A disc containing several large *Minute*+ clones of *sds22 *mutant cells. Mutant cells again shorten to a more cuboidal form with a more linear alignment of nuclei. In addition, several cells have been extruded from the epithelium and are undergoing apoptosis, visible as pyknotic nuclei near the apical or basal surface of the epithelium (arrow). Deep infolding of the mutant tissue causes islands of wild-type tissue to be cut off from surrounding cells. (C) When apoptosis is blocked in *sds22 *mutant cells by expression of the caspase inhibitor p35, mutant clones (marked by expression of RFP) are still extruded from the epithelium. A ball of mutant cells with no epithelial character has dropped basally from the epithelium.

To examine the effect of this mutant more closely, we induced *sds22 *mutant clones in the developing wing imaginal disc, a commonly used model system for studying the growth and morphology of clones of cells. The wing disc is composed of a columnar epithelium, a pseudostratified monolayer, that is continuous with an overlying peripodial epithelium, a squamous monolayer (Fig 1A). We induced GFP negative clones of wild-type (Fig 1A) and *sds22 *mutant (Fig 1B and 1C) cells and allowed the clones to grow for two (Fig 1A and 1B) or three days (Fig 1C). The *sds22 *mutant clones survived and grew similarly to wild-type clones for the first two days (Fig 1A and 1B) but were eliminated by three days (Fig 1C). This phenotype suggests that the *sds22 *mutant cells are sensitive to cell competition, a phenomenon whereby weaker cells are killed off by their more strongly growing neighbours (see, for example, [12]).

We therefore gave *sds22 *mutant clones a growth advantage over their neighbours with the *Minute *technique. This technique slows the growth of cells neighbouring the clone by making these cells heterozygous mutant for a *Minute *gene, encoding a ribosomal subunit. With this method, wild-type clones grow to very large sizes (Fig 1D) after 2 days. In contrast, *sds22 *mutant cells formed smaller clones over the same time period and exhibited defects in epithelial morphology (Fig 1E). After 3 days, the *sds22 *mutant cells were still largely eliminated from the tissue, being extruded basally from the epithelium and undergoing apoptosis (Fig 1F). Extrusion of the *sds22 *mutant cells left behind dramatic folds in the epithelium (Fig 1F), more easily appreciated when the filamentous actin cytoskeleton was visualised by staining with phalloidin (Fig 1G, H & I).

To examine the cellular basis for these phenotypes, we examined confocal cross-sections of discs carrying *sds22 *mutant clones (Fig 2). By examining both smaller, younger clones (Fig 2A; marked by absence of GFP) and larger, older clones (Fig 2B; marked by absence of GFP) we observed a progressively stronger phenotype. In smaller clones, the mutant cells appear abnormally short in their apical-basal axis, adopting a more cuboidal morphology than their pseudostratified columnar neighbours (Fig 2A). Although the mutant cells in these small clones are abnormally short, they retained their polarised epithelial character, with normal localisation of the adherens junction component, Armadillo (Arm; Fig 2A). In larger clones, abnormally short mutant cells were also visible, but, more dramatically, infolding and extrusion of the mutant cells created islands of wild-type epithelium, surrounded by mutant cells (Fig 2B; also visible in Fig 1G, arrow). A large number of pyknotic nuclei, indicating apoptotic cells, were visible in the *sds22 *mutant clones (Fig 2B), some of which appeared to have left the epithelium entirely (Fig 2B, arrow). These results suggest that *sds22 *mutant cells first change shape, adopting a more cuboidal morphology, and later leave the epithelium and apoptose, leaving behind a deep infolding of the tissue.

We next tested whether extrusion of *sds22 *mutant cells from the epithelium was a cause or consequence of their apoptosis. We therefore prevented apoptosis of *sds22 *mutant cells by expression of the baculovirus caspase inhibitor, p35. We found that mutant cells were still extruded from the epithelium, collecting as a ball of round cells on the basal side of the disc (Fig 2C; positively marked by expression of RFP). These results show that *sds22 *is essential to maintain the epithelial integrity of wing imaginal disc cells and suggest that extrusion of mutant cells is the cause of apoptosis.

### *sds22 *is required for epithelial morphology in ovarian follicle cells

**Figure 3:**
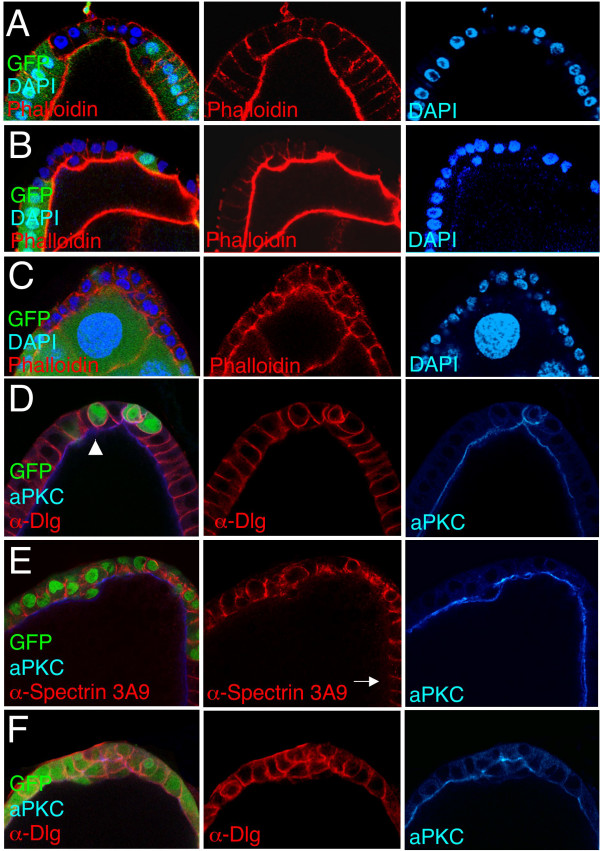
***sds22 *is required to maintain epithelial cell shape and polarity in follicle cells**. (A-C)*sds22 *mutant clones induced in the follicular epithelium of *Drosophila *egg chambers with *hs.Flp *and marked by the absence of GFP. (A) An *sds22 *mutant clone showing altered cell shape. Cells have shortened along their apico-basal axes. Phalloidin (red) marks the filamentous actin cytoskeleton, which can be seen to be slightly weaker along lateral cell membranes within the mutant clone. DAPI (blue) marks nuclei. (B) An *sds22 *mutant clone showing both altered cell shapes and cells beginning to round up and form a double layer. Phalloidin (red) marks cell outlines. DAPI (blue) marks nuclei. (C) An *sds22 *mutant clone showing multi-layering of mutant cells, indicating loss of apico-basal polarity. Phalloidin (red) marks cell outlines. DAPI (blue) marks nuclei. (D-F) *UAS.sds22-IR *and GFP expressing clones stained for anti-aPKC (apical marker, blue) and anti-Dlg or anti-α-Spectrin 3A9 (both baso-lateral markers, red). (D) Small *UAS.sds22-IR *and GFP expressing clone showing spreading of basolateral marker (Dlg, red) around the plasma membrane. Mutant cells have a rounded appearance. Note, only one mutant cell is a polar cell, identified by strong aPKC staining (blue). Other mutant cells (e.g.: arrowhead) show reduced or diffuce aPKC staining. (E) Larger *UAS.sds22-IR *and GFP expressing clone showing spreading of basolateral marker (anti-α-Spectrin 3A9, red) around the plasma membrane, indicating a failure to maintain apical-basal polarity. aPKC staining (blue) remains largely apical in this clone. Arrow points to the absence of anti-α-Spectrin 3A9 staining in wild-type apical membranes. (F) A very large *UAS.sds22-IR *and GFP expressing clone showing spreading of the basolateral maker (Dlg, red) around the plasma membrane. The cells form a double layer. aPKC staining (blue) appears abnormally reduced or diffuse.

To test whether the *sds22 *is specifically required in imaginal discs or more generally required in epithelia, we examined the follicular epithelium of *Drosophila *egg chambers (Fig 3A). As in the wing, *sds22 *mutant clones showed a spectrum of defects in epithelial morphology, depending on clone size. In smaller clones, cells appear abnormally contracted along their apical-basal axis (Fig 3A). In many larger clones, cells rounded up and tended to form double layers (Fig 3B&C). When markers of apical-basal polarity were analysed in *sds22 *mutant clones (not shown) or in clones expressing RNAi against *sds22 *(*UAS.sds22-IR*; Fig 3D, E&F, clones marked by GFP expression), abnormal spreading of baso-lateral markers (anti-Dlg or anti-α-Spectrin 3A9 staining) around the cell into apical regions was detected. In some clones, the apical marker aPKC appeared relatively normal (Fig 3E), while in others aPKC staining appeared reduced and more diffuse (Fig 3D&F). These results indicate that follicle cell epithelia deficient for *sds22 *show both cell shape and polarity defects.

### *sds22 *mutant cells have a defective actin cytoskeleton

**Figure 4:**
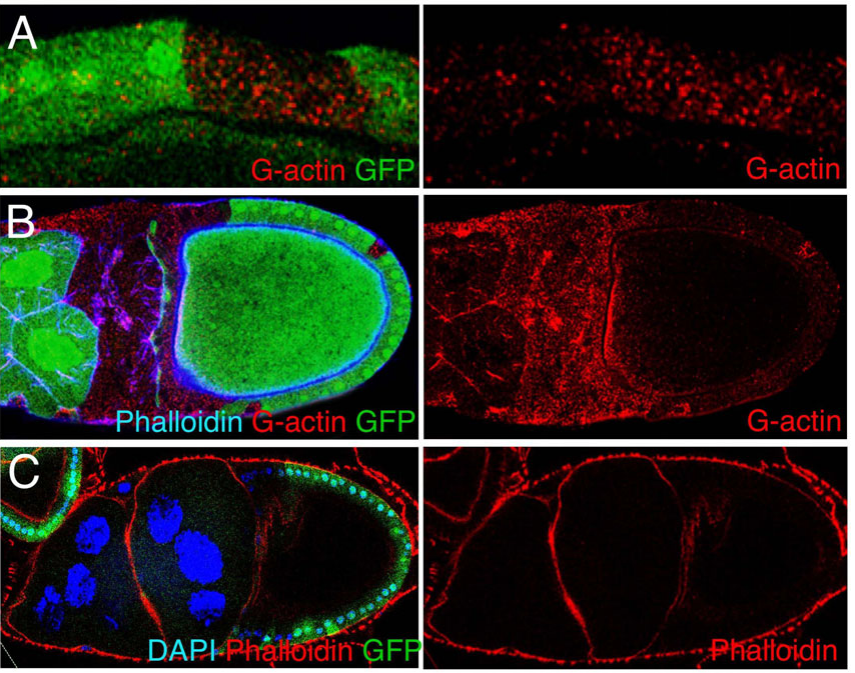
***sds22 *mutant cells accumulate G-actin and exhibit breakdown of F-actin**. *sds22 *mutant clones in ovarian follicle cells and giant nurse cells, marked by the absence of GFP. (A) Globular actin (G-actin, red) is upregulated in *sds22 *mutant clones in follicle cells. (B) Globular actin (G-actin, red) is also upregulated in *sds22 *mutant nurse cells. (C) Nurse cell membranes, lined with filamentous actin (marked by Phalloidin, red), break down in *sds22 *mutant nurse cells, leading to large cells containing multiple nuclei.

We next examined the actin cytoskeleton in *sds22 *mutant cells in egg chambers. Staining with an antibody recognising both monomeric G-actin and filamentous F-actin, we found a striking punctate accumulation of actin in mutant cells in both the follicular epithelium and giant nurse cells (Fig 4A & B). A similar accumulation of F-actin was not observed with Phalloidin staining (Fig 3A, 4C) indicating that G-actin is preferentially accumulating in *sds22 *mutant cells. Notably, in the nurse cells, breakdown of the plasma membrane and underlying cortical actin cytoskeleton led to multinucleated nurse cells (stained with Phalloidin; Fig 4C). Strikingly, these *sds22 *mutant phenotypes, together with the apico-basal contraction phenotype described above, are similar to phenotypes reported for a mutant in the PP1β9c phosphatase [11].

### *sds22 *encodes a highly conserved PP1 phosphatase regulatory subunit

**Figure 5:**
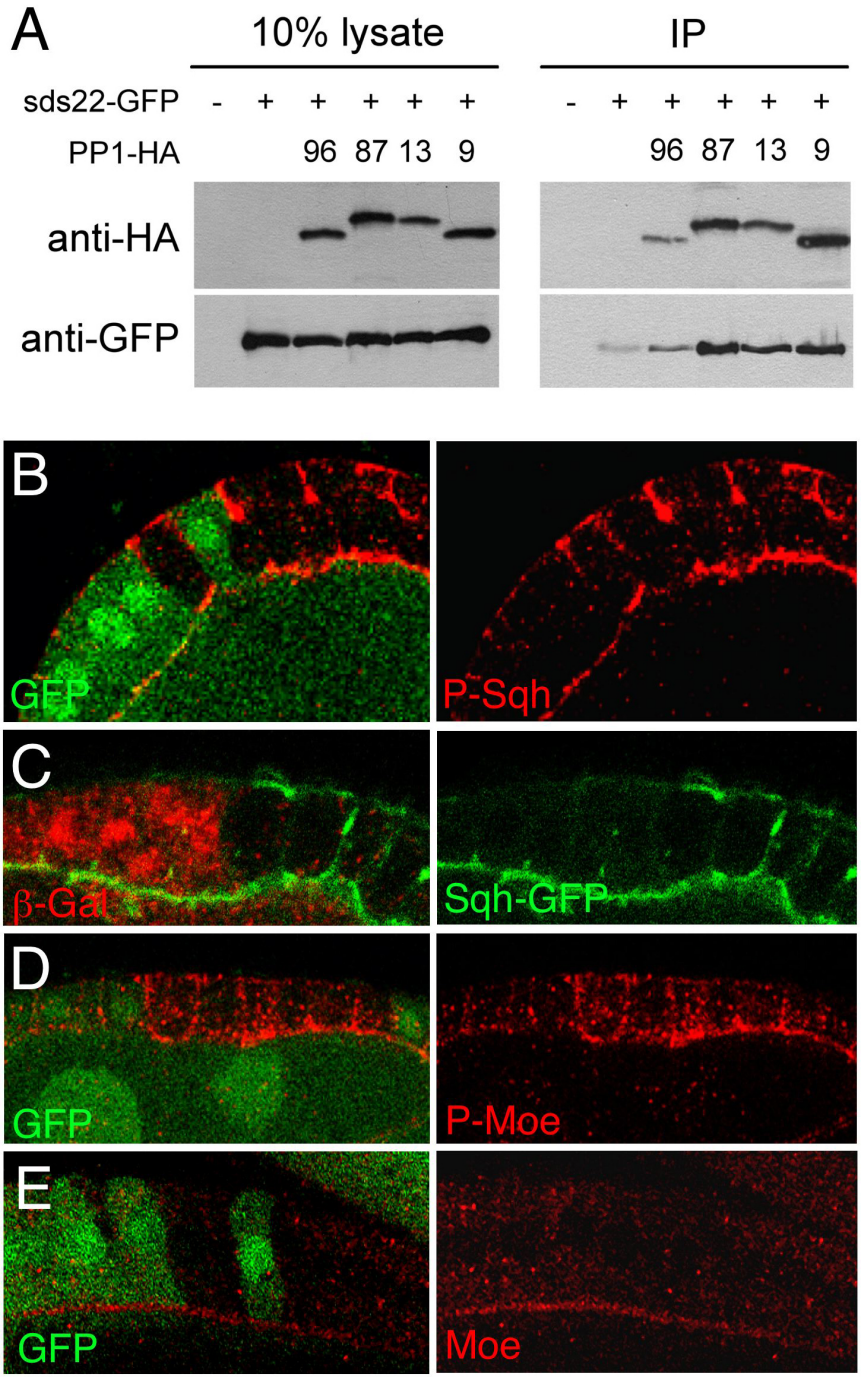
**Sds22 binds to all four *Drosophila *PP1 proteins and *sds22 *mutant cells exhibit hyper-phosphorylation of the PP1 targets Sqh/MRLC and Moe**. (A) Co-immunoprecipitation of Sds22-GFP with each of the four HA-tagged PP1 isoforms (labelled: 96, 87, 13 and 9) from *Drosophila *S2 cell lysates. Beads conjugated to anti-HA antibodies were used to precipitate each of the HA-tagged PP1s and Sds22-GFP was found to efficiently co-precipitate. Note that, in the absence of transfected HA-tagged PP1, only a tiny amount of Sds-22 protein precipitates with the HA-beads. (B) Phosphorylated Sqh/MRLC (P-Sqh, red) is elevated and spreads basally in *sds22 *mutant clones (marked by absence of GFP, green). (C) Levels of total Sqh/MRLC protein (monitored by a *Sqh-GFP *transgene, green; [27] are not altered in *sds22 *mutant clones (marked by absence of beta-Gal, red). (D) Phosphorylated Moesin (P-Moe, red) is elevated and spreads basally in *sds22 *mutant clones (marked by absence of GFP, green). (E) Overall protein levels of Moesin (Moe, red) do not increase in *sds22 *mutant clones (marked by absence of GFP, green).

Experiments in yeast and mammalian cells suggest that Sds22 binds to and regulates PP1 phosphatases. We therefore tested whether this is also the case for *Drosophila *Sds22. *Drosophila *has four PP1 phosphatases, named after their isotype and cytological location: PP1 α96a, β87b, β13c and β9c. We expressed HA-tagged versions of each of these PP1s with GFP-tagged Sds22 in *Drosophila *cells and subjected cell lysates to immunoprecipitation with anti-HA antibodies (Fig 5A). GFP-tagged Sds22 was efficiently co-precipitated when any of the four HA-tagged PP1s was co-expressed. In the absence of HA-tagged PP1s, only a small background level of GFP-Sds22 associated with HA-beads. The results indicate that Sds22 binds to PP1s, confirming results in yeast and mammalian cells.

### Phosphorylation of two potential PP1 targets, Sphaghetti Squash and Moesin, is increased in *sds22 *mutant cells

We next sought evidence that Sds22 is required for PP1 phosphatase activity in *Drosophila*. Previous work suggested that Sqh/MRLC is a target of PP1 β9c, because levels of phospho-Sqh are elevated in *PP1 β9c *mutant cells [9,11]. In addition, evidence from mammalian cells suggested that the same phosphatase that targets Myosin II regulatory light chain may also target Moesin (Moe), the sole *Drosophila *ERM protein [13]. We therefore examined levels of phospho-Sqh and phospho-Moe in *sds22 *mutant cells by immunostaining with phospho-specific antibodies. Levels of both phospho-Sqh (Fig 5B) and phospho-Moe (Fig 5D) were elevated relative to total Sqh (Fig 5C) and Moe (Fig 5E) in *sds22 *mutant cells. Note that the excess phospho-Sqh and phospho-Moe staining accumulates on both apical and baso-lateral membranes in mutant cells (in contrast to their apical concentration in wild-type cells). Thus, *sds22 *is required to restrict the phosphorylation of both Moe and Sqh/MRLC.

### Conserved function of Sds22 in human cells

**Figure 6:**
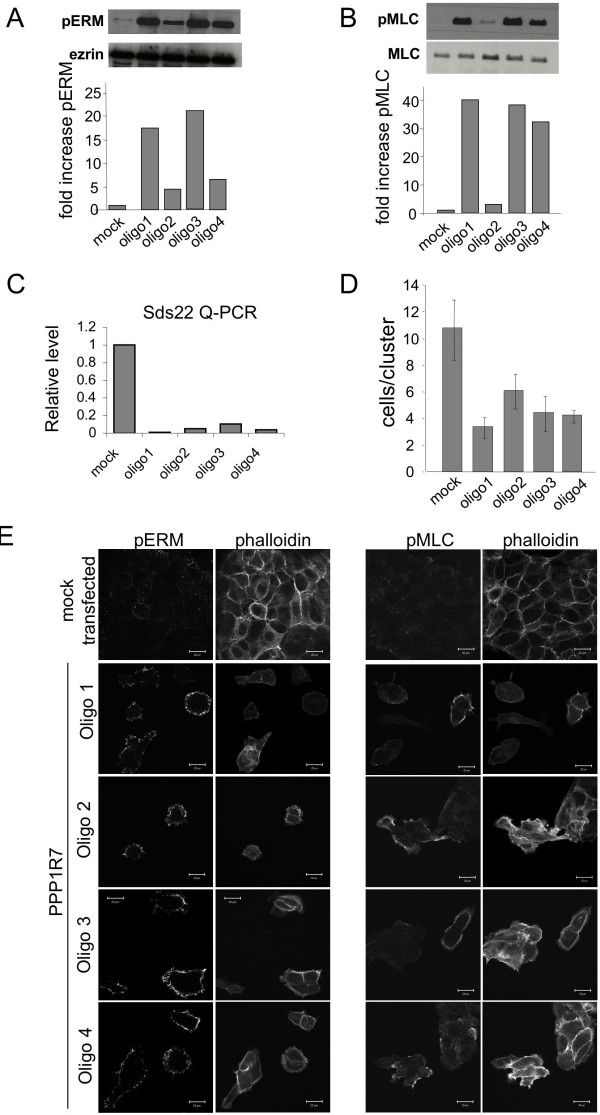
**The function of Sds22 is conserved in human cells**. The human homologue of Sds22, PPP1R7, was depleted with four siRNA oligos in the human A431 squamous cell carcinoma line. (A) Western blot showing changes in ERM protein phosphorylation and total Ezrin levels in control and PPP1R7 siRNA transfected A431 cells. Bar chart shows fold changes in p-ERM levels normalized to total Ezrin levels in control and PPP1R7 transfected A431 cells. (B) Bar chart shows fold changes in p-S19-MLC levels normalized to total MLC levels in control and PPP1R7 transfected A431 cells. (C) RT-PCR showing beta-actin and PPP1R7 levels in control and PPP1R7 siRNA transfected A431 cells. Bar chart shows % changes in PPP1R7 levels normalized to beta-actin levels. (D) Bar graph showing the average number of cells per cluster in control and PPP1R7 transfected A431 cells. (E) Representative images of p-ERM and p-S19-MLC staining in control and PPP1R7 transfected A431 cells.

Sds22 is widely conserved in the animal kingdom, including a mammalian homologue PPP1R7. We therefore investigated if PPP1R7 performed similar functions in mammalian cells to Sds22 in *Drosophila*. A431 cells are keratinocytes that retain adherens junctions and grow in 'quasi-epithelial' clusters despite being transformed. First, we investigated the morphology of A431 cells lacking PPP1R7. Control A431 cells formed colonies with an average of 10 cells (Fig 6D&E) whereas cells depleted for PPP1R7 formed smaller clusters usually consisting of 4–6 cells (Fig 6D&E). Thus, PPP1R7 helps to maintain the cohesion of groups of human cells of epithelial origin.

We next tested whether the PPP1R7 knockdown phenotype reflected a conserved function in regulating the phosphorylation status of Ezrin, Radixin and Moesin (hereafter refered to as ERM proteins) and Myosin Light Chain (MLC). Depletion of PPP1R7 with siRNA oligos caused a dramatic increase in the phosphorylation of both ERM proteins and MLC (on Ser19; Fig 6A, B & E). This striking result indicates that the biochemical function of Sds22/PPP1R7 is conserved between Drosophila and mammals. To verify the RNAi effect, we measured depletion of PPP1R7 mRNA levels in A431 cells by quantitative RT-PCR (Fig 6C). The mild variations observed between the extent of knockdown and phenotypic strength with different siRNA oligos are likely to result from experimental differences between the staining and PCR.

## Discussion

Our results show that Sds22, a protein previously identified as a PP1 phosphatase regulatory subunit in yeast, is essential for PP1 function in *Drosophila *tissues. We have also shown that the phosphorylation state of both Moesin and Sqh/MRLC depends upon the Sds22/PP1 phosphatase. Our experiments do not prove that Moesin and Sqh/MRLC are direct substrates of Sds22/PP1, but this is a distinct possibility, as previous work has shown that Sqh/MRLC can be found in a complex with PP1. We have shown that this function of Sds22 is conserved in mammalian cells, consistent with previous biochemical evidence that PPP1R7 (the mammalian Sds22 homologue) binds to PP1 proteins.

Moesin and Sqh/MRLC are key regulators of the actin cytoskeleton. Our work favours a model in which restricted activation of Moesin and Sqh/MRLC maintains an apical contractile bundle of actin filaments that is essential for the shape and integrity of epithelial cells. Both Moesin and Sqh/MRLC are activated by phosphorylation at the apical membranes of epithelia. In the case of Moesin, this phosphorylation is essential for epithelial integrity and depends on the apically localised kinase Slik [10]. In the case of Sqh/MRLC, this phosphorylation is essential for cells to maintain their columnar shape and depends on Rho kinase [9]. Our work indicates that this restricted activation of these proteins at the apical membrane is complemented by dephosphorylation of Moesin in other parts of the cell by ubiquitous PP1 phosphatases containing the regulatory subunit Sds22. In *sds22 *mutants, these actin regulators are activated along the entire cell membrane at high levels, leading to an abnormal contraction of the cells along their apical-basal axis. A similar, but milder, phenotype is visible in *PP1β9c *mutant clones [11] and in clones overexpressing a phospho-mimetic form of Sqh/MRLC [14,15].

A second phenotype observed upon depletion of Sds22 is a loss of apical-basal polarity, associated with cells rounding up and forming multi-layers. Baso-lateral polarity markers appeared to be more strongly affected than apical markers in follicle cell epithelia. In imaginal discs, loss of polarity in *sds22 *mutant clones would explain the extrusion and apoptosis of these cells. Importantly, both the loss of polarity in follicle cells and the extrusion of imaginal disc cells were most commonly visible in larger, older clones, indicating that this phenotype takes longer to manifest than the abnormal cell shape phenotype. This raises the possibility that Sds22 may not be a direct regulator of cell polarity, but rather is required for polarity as a consequence of its regulation of Sqh/MRLC and Moesin. Alternatively, there may be additional targets of Sds22 that regulate the localisation of polarity markers.

### Evolution of Sds22 and PP1 functions

*sds22 *was first identified in the single-celled yeast, *Schizosaccharomyces pombe *where it encodes a nuclear protein that directly binds to and regulates a PP1 phosphatase [16,17]. In *S. pombe*, Sds22 is essential for this phosphatase to control events during mitosis [16,17]. In metazoans, the PP1 family has expanded and Sds22 has aquired additional functions in cell shape and polarity. Accordingly, in both *Drosophila *and mammals, Sds22 is not exclusively localised to the nucleus and is instead found throughout the cell (Additional Fig 3; [18,19]).

### Discrepancies between *sds22 *and *PP1 *mutant phenotypes in *Drosophila*

*Drosophila *have four PP1 phosphatases, named after their isotype and cytological location:*PP1α13c*,*PP1α87c*, *PP1α96a *and *PP1β9c*. The potential for redundancy among these four genes complicates genetic analysis. Nevertheless, mutation of individual *PP1 *genes indicates that some may have unique functions. Mutant alleles of PP1α87c, which contributes 80% of the total PP1 phosphatase activity [20], show strong defects in mitosis [21]. Of the other three *Drosophila *PP1s, only one is essential, PP1β9c. Interestingly, the morphological phenotype of *PP1β9c-*mutant cells resembles that of *sds22 *mutant cells in both follicle cells and nurse cells [11]. In *PP1β9c *mutant clones of the follicular epithelium, cells are shortened in their apical-basal axis and cytosolic levels of G-actin and phospho-Sqh are increased [11]. However, *PP1β9c *mutant cells were not reported to show defects in cell polarity.

Why do *sds22 *mutants not exhibit the mitotic defects found *PP1α87c *mutants? A likely explanation is that our *sds22 *mutant allele is not a null. In fact, depletion of *sds22 *by RNAi in *Drosophila *cells was recently reported to cause mitotic defects [22]; [D. Glover, personal communication]. Thus, our *sds22 *allele may be a hypomorph that reveals the function of Sds22 in regulating cell shape and polarity.

Why do *PP1β9c *mutants not exhibit the polarity defects found in *sds22 *mutants? A plausible explanation is that the presence of the three other PP1s masks this phenotype. *PP1α87c *mutants, which remove 80% of PP1 activity, might therefore be expected to show a polarity defect. However, the mitotic defects in these mutants prevent growth of clones, obscuring any potential polarity defect. Thus, *sds22 *mutations are a convenient way of modulating PP1 activity that can reveal otherwise hidden phenotypes.

Finally, other proteins have been identified which link PP1 phosphatases to target proteins in *Drosophila*. These include MYPT-75D, which appears to recruit Sqh to PP1 and its loss has a weaker phenotype than loss of Sds22 [11]. Another MYPT is MBS, whose loss of function phenotype even more strongly resembles that of Sds22 [23]. While these two related MYPTs may be partially redundant with one another, it is unlikely that they are also redundant with Sds22. Redundancy between Sds22 and MYPTs would likely result in Sds22 being non-essential. Further experiments will be necessary to establish whether the Sds22/PP1 complex and MYPT/PP1 complexes act independently or together in cells.

## Conclusion

We have confirmed work in other organisms by showing that Sds22 acts as an essential subunit of PP1 phosphatases in both *Drosophila *and mammals and controls cell shape and polarity in epithelial cells. There is an interesting parallel between the *sds22 *mutant phenotype in *Drosophila *and the escape of metastasising tumour cells from epithelia to invade local tissues. Interestingly, PPP1R7 has been reported to be significantly downregulated in human squamous cell carcinomas (HNSCCs) and other cancers, such as melanoma and prostate cancer . Thus, downregulation of Sds22/PPP1R7 may contribute to tumour progression in humans.

## Methods

### Generation of mutant *sds22 *alleles

The PiggyBac transposon based mutagenesis screen has been described [24,25]. PiggyBac insertion sites were mapped by inverse PCR. The PiggyBac is contains a gene-trap module designed to prevent translation of downstream genes. Excision of the PiggyBac insertion in *sds22*^PB1173 ^was achieved by introduction of the PiggyBac transposase.

### Molecular Biology

*sds22 *cDNA was amplified by PCR from the GM06266 cDNA clone (Berkley Drosophila Genome sequencing project), cloned into a pEGFP-N1 vector (Clontech Laboratories) to create a C-terminal GFP fusion, and then subcloned into a pUAST expression vector [26] for P-element transformation.

### Fly genetics

The *tubulin.Gal4, c204.Gal4 (BL3751) *and *engrailed.Gal4 *fly lines are described in Flybase and available from the Bloomington stock centre.

Clones of genetically marked homozygous *sds22 *mutant cells were generated using the following genotypes:

*yw ey.flp/+; FRT82 Minute Ubi.GFP/FRT80 82 sds22*^PB1173 ^(eye discs)

*w hsp70.flp/+; FRT82 Ubi.GFP/FRT80 82 sds22*^PB1173 ^(wing discs, eye discs and egg chambers)

*w hsp70.flp/+; FRT82 Minute Ubi.GFP/FRT80 82 sds22*^PB1173 ^(wing discs, eye discs and egg chambers)

*w hsp70.flp/+;Sqh.GFP/+; FRT82 arm.lacZ/FRT80 82 sds22*^PB1173 ^(*Sqh-GFP *gift of R. Karess; egg chambers)

*w hsp70.flp UAS.RFP/+; tub.Gal4/UAS.p35; FRT80 82 sds22*^PB1173^/*FRT82 tub.Gal80 *(the MARCM system for expressing p35 in mutant clones in wing discs)

*yw ey.flp UAS.GFP; tub.Gal4/+; FRT80 82 sds22*^PB1173^/*FRT82 tub.Gal80 *(the ey.flp-MARCM system for eye discs)

*yw ey.flp UAS.GFP; tub.Gal4/UAS.Sds22-GFP; FRT80 82 sds22*^PB1173^/*FRT82 tub.Gal80 *(transgenic rescue with the ey.flp-MARCM system)

For imaginal wing discs, *hs.flp *clones were generated by heat shocking larvae at 37°C for 1 hr at 60 ± 12 hrs or 84 ± 12 hrs of development. For egg chambers, third instar larvae were heat shocked for 1 hr at 37°C. Adults were fattened on yeast for 2 days prior to dissection.

The *UAS.sds22-IR *transgenic RNAi line was obtained from the Vienna Drosophila RNAi Centre (VDRC, transformant number 11788) and driven in clones by induction of Gal4 with the 'flip-out' system in females of the following genotype:

hs.flp; actin.FRT.y STOP.FRT.Gal4 UAS.GFP/UAS.sds22-IR

### Immuno-fluorescence in *Drosophila*

Imaginal discs and egg chambers were dissected on ice, fixed in 4% formaldehyde for 20 min and incubated with primary antibodies overnight at 4°C in BBT (PBS, 0.2% TritonX-100, 0.1% BSA; for the α-Sqh antibody: PBS, 0.3% Tween-20, 0.1% BSA). Secondary antibodies, Phalloidin and DAPI were added for 2 hrs at room temperature. The following antibodies and chemicals were used: rabbit α-aPKC (1:200; Santa Cruz Biotech), mouse α-alpha-Spectrin (1:50; Developmental Studies Hybridoma Bank, DSHB), mouse α-Dlg (1:100; DSHB), mouse α-Armadillo (1:10; DSHB), rabbit α-actin (1:100; preferentially stains G-actin; Sigma Aldrich), rabbit α-betaGal (1:100; Cappel) rabbit α-Moesin (1:100; a gift from D. Kiehart), rabbit α-Phospho-Moesin (1:100; Cell Signaling Technologies), rabbit α-Phospho-MRLC (1:500; a gift from D. Bennett), 488_Phalloidin and 568_Phalloidin (1:1000; Molecular Probes, Invitrogen), DAPI (1:1000; Sigma Aldrich). As secondary antibodies, goat α-rabbit Cy5 and goat α-rat Cy5 (1: 200; Jackson Immuno Research laboratories) were used. Images were taken with a Leica TCS SP2 Confocal Microscope.

### Immuno-precipitation

*Drosophila *S2 cells were transfected with combinations of Sds22-GFP and HA-tagged PP1 proteins, expressed from the pUAST vector by co-transfection of the Copper-inducible pMT-Gal4 plasmid. 2 days after induction with 700 μM CuSO_4_, cell lysates were harvested and incubated overnight at 4 degrees with agarose beads conjugated to mouse anti-HA antibodies. The beads were spun down and washed several times. Precipitated proteins were eluted from the beads by boiling in SDS-sample buffer and then analysed by western blotting with rat anti-HA and rabbit anti-GFP antibodies.

### Cell culture and siRNA Transfection

A431 cells were grown in DMEM supplemented with 10% FCS. A431 cells were transfected using OligofectamineTM Reagent (invitrogen #12252-011). Briefly, cells were plates at 60% confluence and subjected to transfection the following day using 100 nM final concentration of siRNA. siRNA against PPP1R7 was purchased from Dharmacon and sequences are: oligo 1: ggacagagaugcagaggauuu (D-019589-01), oligo 2: uaacagagcuggagauucuuu (D-019589-02); oligo3: gaaaauaucagccaucuaauu (D-019589-03); oligo 4: gacauugcaucaaauagaauu (D-019589-04). Transfections were stopped after 48 h.

### Immuno-fluorescent staining of cultured cells

A431 cells were fixed using 4% PFA in PBS for 15 minutes at room temperature, washed with PBS and permeabilised with 0.2% Triton X100 in PBS for 15 minutes. They were blocked with 5%FBS in PBS prior to incubation with pERM or pS19MLC antibodies (Cell Signaling #3141 and #3671 respectively) or TRITC-phalloidin (Sigma #P1951).

### Western blotting

Cells lysates were analysed using 15% SDS-PAGE gels followed by western blotting using pERM, ezrin, pMLC or MLC antibodies (Cell Signalling #3141, #3145, #3671 and #3672 respectively)

### RT-PCR

RNA was isolated from cells using RNeasy Mini kit fron Qiagen. cDNA was synthesized using random primers and M-MLV RT H(-) (Promega). Briefly, 2 μg RNA was mixed with 1 μg random primers. RNAase free water was added to 14 μl. The mixed was heated to 75°C for 5 min and cooled on ice for additional 5 min. 5 μl M-MLV 5× reaction buffer, 5 μl nucleotide pool and 1 μl M-MLV reverse transcriptase H(-) were added. The mixed was incubated at room temperature for 10 min and at 40°C for 50 min. After this, RNAase free water was added to a final volume of 100 μl. Quantitative real-time PCR was carried out according to manufacturer instructions using Platinum^® ^SYBR^® ^Green qPCR SuperMix UDG from Invitrogen (#11744-500) on a Chromo 4 detector (MJ Research). The primers used for actin as control were: (5'-ctgacggccaggtcatca-3', 5'-agaccaaaagccttcatacatc-3') and for PPP1R7: (5'-catcgaaggggttgacaagt-3', 5'-ccccaaaaacaaactctcca-3')

## Authors' contributions

FAG performed the fly experiments, CH performed the human cell experiments, GF examined the RNAi phenotypes in flies and HHS identifed the original fly phenotype. BJT and ES conceived the experiments and wrote the manuscript.

## Supplementary Material

Additional Figure 1 **A genetic screen for genes regulating epithelial morphology recoveres *sds22***. The principle of a genetic screen, based on the *eyeless.Flp (ey.Flp) *system is shown. Homozygous mutant eyes were generated in otherwise heterozygous animals. This screen made use of *Minute *(or alternatively, *cell lethal*) mutations to ensure that large homozygous mutant clones occupied the entire eye (see methods). (A) Viable and phenotypically normal flies were generated when eyes were homozygous for control FRT chromosomes. (B) Pupal lethality was observed when eyes were homozygous mutant for the *sds22 *gene, caused by insertion of a piggyBac transposon (PB1173). (C) Cell lethal mutations allowed development of viable flies with tiny or absent eyes. (D) A third instar eye imaginal disc generated with the *ey.Flp Minute *system showing that cells homozygous for a control FRT chromosome (marked by absence of GFP) occupy almost the entire eye. (E) A third instar eye imaginal disc that is composed largely of *sds22*^PB1173 ^mutant cells (marked by absence of GFP), generated with the *ey.Flp Minute *system. The morphology of the eye disc epithelium is severely disturbed. (F) A third instar eye imaginal disc generated with the *ey.Flp Minute *system using an FRT *cell lethal *chromosome. Most eye cells are eliminated, while the antennal portion of the disc is unaffected. Click here for file

Additional Figure 2 **Transgenic rescue of the *sds22 *phenotype**. The *ey.Flp MARCM *system was used to induce clones of GFP positive cells in the *Drosophila *eye imaginal disc (see methods for genotypes). (A) Control clones of homozygous wild-type cells produce a normal disc morphology. (B) Clones homozygous mutant for *sds22 *severely disrupt disc morphology, causing many folds in the epithelium. (C) Clones homozygous mutant for *sds22 *that also express *UAS.Sds22-GFP *are rescued. Normal disc morphology is restored. Click here for file

Additional Figure 3 **Subcellular localisation of an Sds22-GFP fusion protein**. (A) Confocal section of follicle cell epithelium expressing *UAS.Sds22-GFP *under the control of *c204.Gal4 *and stained for phalloidin (red). Sds22-GFP (green) is found in both nucleus and cytoplasm. (B) Confocal X-Z section of third instar wing disc expressing Sds22-GFP (green) in the posterior compartment under the control of the *en.Gal4 *driver. Sds22-GFP is found in both nucleus and cytoplasm. Click here for file
