# Supplementary figures and images for: Sds22, a PP1 phosphatase regulatory subunit, regulates epithelial cell polarity and shape [Sds22 in epithelial morphology]
Source: BMC Dev Biol. 2009 Feb 19;9:14. doi: 10.1186/1471-213X-9-14 (PMC2652452; doi:10.1186/1471-213X-9-14)

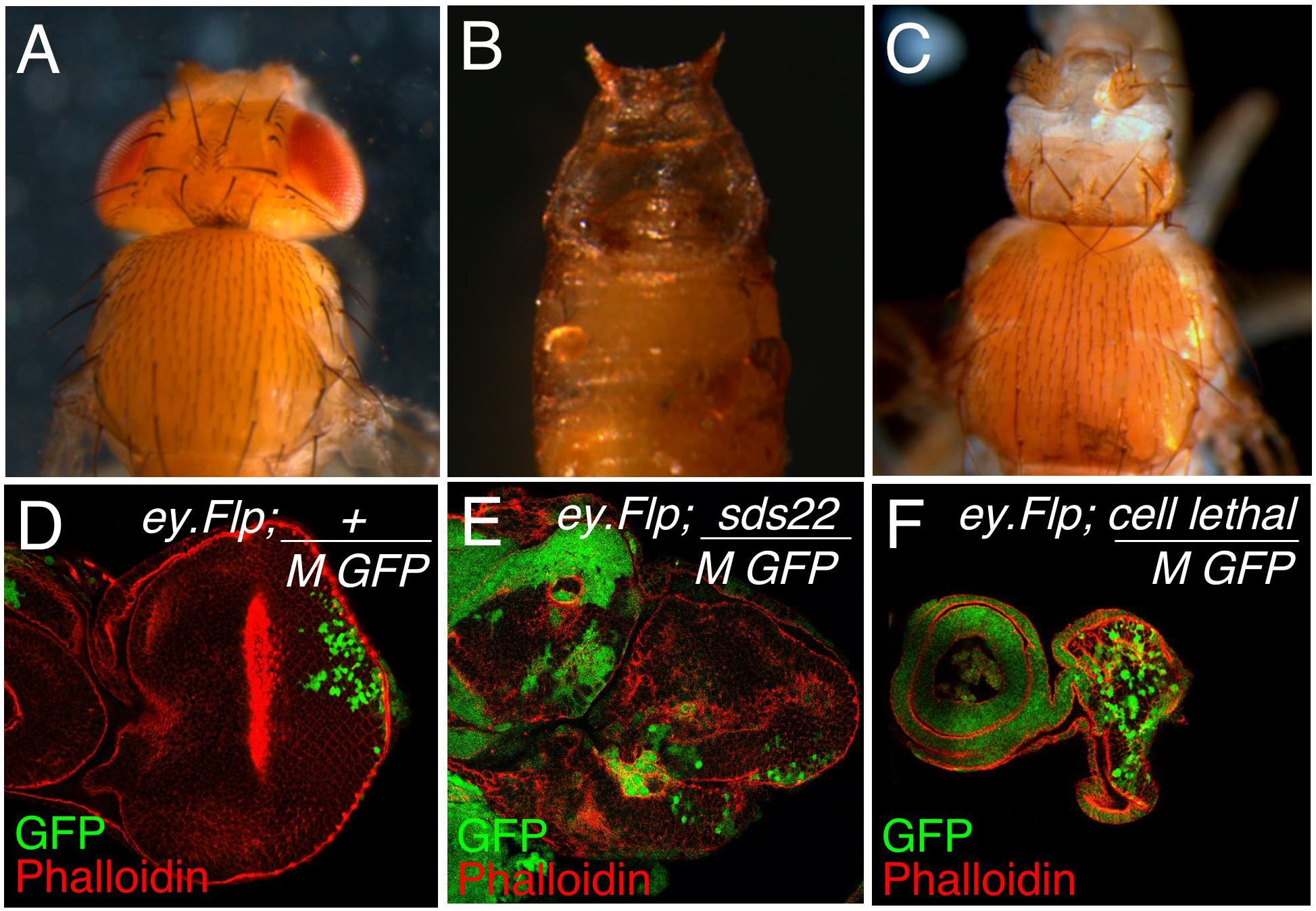

Supplement: Additional Figure 1 — A genetic screen for genes regulating epithelial morphology recoveres sds22. The principle of a genetic screen, based on the eyeless.Flp (ey.Flp) system is shown. Homozygous mutant eyes were generated in otherwise heterozygous animals. This screen made use of Minute (or alternatively, cell lethal) mutations to ensure that large homozygous mutant clones occupied the entire eye (see methods). (A) Viable and phenotypically normal flies were generated when eyes were homozygous for control FRT chromosomes. (B) Pupal lethality was observed when eyes were homozygous mutant for the sds22 gene, caused by insertion of a piggyBac transposon (PB1173). (C) Cell lethal mutations allowed development of viable flies with tiny or absent eyes. (D) A third instar eye imaginal disc generated with the ey.Flp Minute system showing that cells homozygous for a control FRT chromosome (marked by absence of GFP) occupy almost the entire eye. (E) A third instar eye imaginal disc that is composed largely of sds22PB1173 mutant cells (marked by absence of GFP), generated with the ey.Flp Minute system. The morphology of the eye disc epithelium is severely disturbed. (F) A third instar eye imaginal disc generated with the ey.Flp Minute system using an FRT cell lethal chromosome. Most eye cells are eliminated, while the antennal portion of the disc is unaffected. [file 1471-213X-9-14-S1.jpeg]

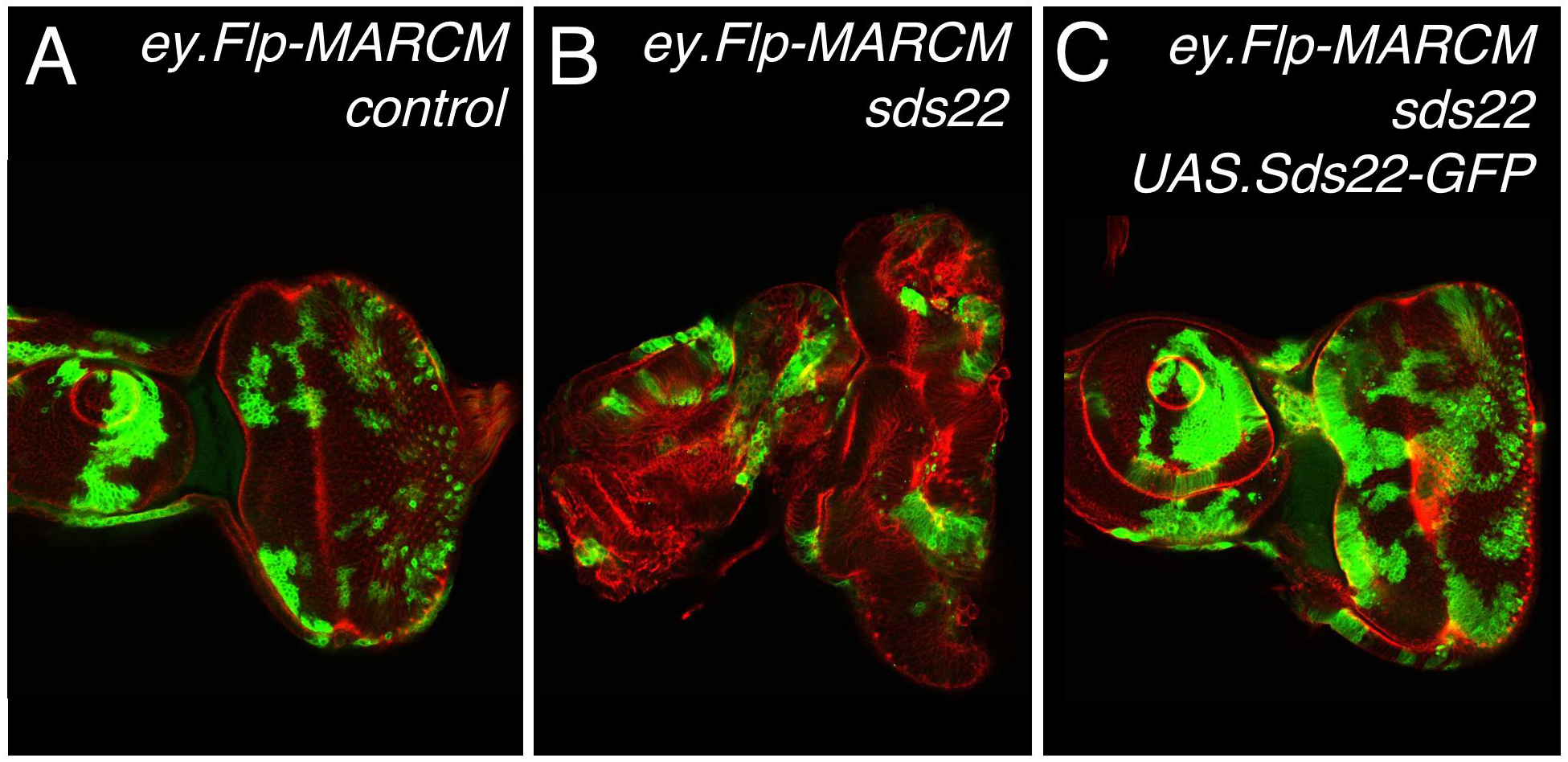

Supplement: Additional Figure 2 — Transgenic rescue of the sds22 phenotype. The ey.Flp MARCM system was used to induce clones of GFP positive cells in the Drosophila eye imaginal disc (see methods for genotypes). (A) Control clones of homozygous wild-type cells produce a normal disc morphology. (B) Clones homozygous mutant for sds22 severely disrupt disc morphology, causing many folds in the epithelium. (C) Clones homozygous mutant for sds22 that also express UAS.Sds22-GFP are rescued. Normal disc morphology is restored. [file 1471-213X-9-14-S2.jpeg]

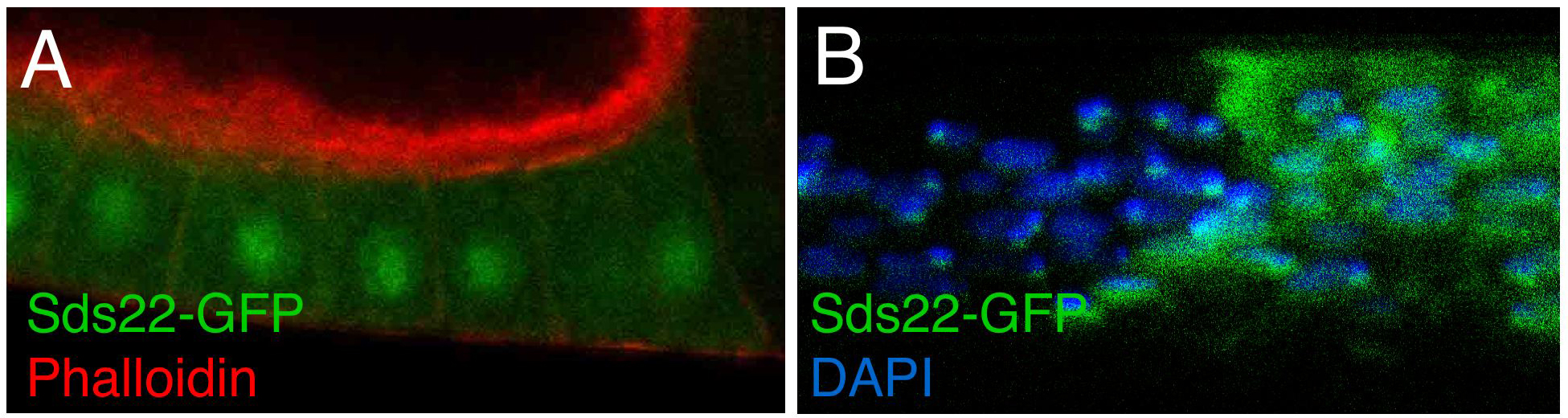

Supplement: Additional Figure 3 — Subcellular localisation of an Sds22-GFP fusion protein. (A) Confocal section of follicle cell epithelium expressing UAS.Sds22-GFP under the control of c204.Gal4 and stained for phalloidin (red). Sds22-GFP (green) is found in both nucleus and cytoplasm. (B) Confocal X-Z section of third instar wing disc expressing Sds22-GFP (green) in the posterior compartment under the control of the en.Gal4 driver. Sds22-GFP is found in both nucleus and cytoplasm. [file 1471-213X-9-14-S3.jpeg]
